# Clinical and Occupational Predictors of Mortality in Ebola Virus Disease: A Commentary from the Democratic Republic of Congo (2018–2020)

**DOI:** 10.3390/idr17030071

**Published:** 2025-06-18

**Authors:** Jean Paul Muambangu Milambo, Charles Bitamazire Businge

**Affiliations:** Department of Gynaecology and Obstetrics, Faculty of Medicine and Health Sciences, Walter Sisulu University, Mthatha Campus, Mthatha 5099, South Africa

**Keywords:** Ebola virus disease, Democratic Republic of Congo, clinical outcomes, outbreak policy, health system resilience, mortality, public health preparedness

## Abstract

**Background:** This commentary analyzes demographic, clinical, and occupational characteristics associated with Ebola virus disease (EVD) outcomes during the 2018–2020 outbreak in the Democratic Republic of Congo (DRC). **Methods:** A total of 3477 EVD cases were included. Descriptive statistics and univariate and multivariate Cox regression analyses were performed to evaluate associations between clinical outcomes and patient characteristics. Comorbidity estimates and healthcare worker (HCW) occupational exposure data were incorporated based on the literature. **Results:** The median age was 26.5 years (SD = 16.1), with the majority (59.7%) aged 20–59. Males represented 51.3% of the cohort. Most patients (81.8%) worked in occupations that were not disease-exposing. Overall, 450 patients (12.9%) died. Although comorbidities initially appeared predictive of mortality (unadjusted HR: 3.05; 95% CI: 2.41–3.87), their effect was not statistically significant after adjustment (adjusted HR: 1.17; 95% CI: 0.87–1.59; *p* = 0.301). The strongest predictor of death was clinical status at admission: patients classified as “very sick” had an alarmingly high adjusted hazard ratio (HR) of 236.26 (95% CI: 33.18–1682.21; *p* < 0.001). Non-disease-exposing occupations were also associated with increased mortality (adjusted HR: 1.75; 95% CI: 1.33–2.31; *p* < 0.001). **Conclusions:** Despite improvements in outbreak response, mortality remains disproportionately high among patients presenting in critical condition and those outside the health sector. These findings underscore the importance of early detection strategies and enhanced protection for all occupational groups during EVD outbreaks.

## 1. Introduction and Rationale

The Democratic Republic of Congo (DRC) has endured persistent and recurrent Ebola virus disease (EVD) outbreaks since the virus was first identified in Yambuku in 1976. Between 2014 and 2022, the DRC experienced several waves of EVD, each presenting immense challenges to the country’s fragile health system. These outbreaks have not only led to high mortality but also revealed systemic weaknesses in outbreak preparedness, surveillance, and health service delivery [1].

While clinical and virological research has advanced our understanding of EVD pathophysiology and treatment, there has been limited reflection on how repeated outbreaks have cumulatively impacted clinical outcomes and system-level response [1,2,3,4]. The broader policy and public health implications—particularly in terms of patient management, mortality predictors, and health system adaptation—remain underexplored.

This commentary is based on field experience, expert observation, and secondary analysis of national surveillance data. It offers insights into how the DRC’s health system has responded to recurring EVD challenges and the implications of these responses for clinical outcomes. It aims to stimulate policy dialogue and support evidence-informed decisions for future epidemic preparedness. This commentary examines the demographic, clinical, and occupational characteristics associated with Ebola virus disease (EVD) outcomes during the 2018–2020 outbreak in the Democratic Republic of the Congo (DRC).

## 2. Hypothesis and Aim

We hypothesize that the cumulative effect of recurrent EVD outbreaks has led to a shift in clinical outcomes, which have improved in some areas due to enhanced management practices, yet persistently face challenges from contextual vulnerabilities such as late presentation and limited access to care.

**Objective:** This study aims to examine the impact of recurrent EVD outbreaks on clinical outcomes between 2018 and 2022 in the DRC, and to identify key factors influencing mortality and survival, thereby informing policy and practice.

## 3. Research Question

What insights can be drawn from available data and field observations on how demographic characteristics, occupational risk, and clinical presentation have influenced survival during recurrent Ebola outbreaks in the DRC?

## 4. Methods and Study Design

This commentary employs a retrospective country profile design, utilizing secondary data collected through the national epidemic surveillance system during the 2018–2022 Ebola virus disease (EVD) outbreaks in the Democratic Republic of the Congo (DRC). Rather than conducting a primary analytical study, the approach synthesizes field-based observations, descriptive statistics, and literature-informed estimates to highlight key demographic, clinical, and occupational trends. Given the operational constraints in conflict-affected regions such as North Kivu, this design enables a contextualized reflection on national response patterns, mortality predictors, and health system challenges. The aim is to inform policy dialogue and support the generation of future analytical research in outbreak-prone settings.

This commentary utilizes retrospective insights from secondary data obtained through the National Epidemic Response Surveillance System in the DRC. The dataset included 3477 confirmed and clinically managed EVD cases from outbreaks between 2018 and 2022.

Though not a formal cohort study, descriptive and inferential analyses were performed to inform policy-relevant reflections. Variables considered included age, gender, occupation, comorbidity, and health status at presentation.

Given the limited availability of demographic and occupational details, the commentary focuses on key variables most relevant to mortality outcomes. The analysis used SPSS v25 for basic statistical interpretation, including logistic regression and Cox proportional hazard models.

## 5. Study Setting

The data reflect patients from multiple outbreak-affected regions, with a majority (91.2%) residing in Nord Kivu, one of the most impacted provinces. Nearly all cases (97.8%) originated from urban settings, reflecting both outbreak epicenters and referral patterns.

## 6. Ethical Considerations

The analysis was approved by the National Epidemic Surveillance Directorate (DES) of the DRC and authorized by the General Directorate of Disease Response. A waiver of informed consent for secondary data analysis was granted by the National Ethics Committee of the Department of Health in the Democratic Republic of the Congo."All data were anonymized and managed following ethical standards. The study was conducted according to the guidelines of the Declaration of Helsinki and approved by the National Ethics Committee of the Ministry of Health of the DRC (n°121/CNES/BN/PMMF/2019). The protocol was approved on 1 June 2019. 

## 7. Results and Interpretive Insights

### 7.1. Patient Characteristics

A total of 3477 Ebola virus disease (EVD) cases were included in the analysis. The median age was 26.5 years (SD = 16.1), with 59.7% (2076/3477) of patients aged between 20 and 59 years. Children under 12 years accounted for 21.5% of cases, adolescents aged 13–19 for 14.4%, and adults aged 60 and above for 4.4%. Slightly more than half of the cohort were male (51.3%).

The majority of patients (81.8%) were engaged in non-disease-exposing occupations, while only 18.2% had roles directly involving contact with Ebola patients (e.g., healthcare providers, burial team members). Most cases originated from urban areas (97.8%), with over 91% located in Nord Kivu Province. Table 1 provides the socio-demographic and clinical characteristics of Ebola cases by outcome.

### 7.2. Survival Outcomes by Demographics and Occupation

Overall, 3027 patients survived (87.1%), while 450 (12.9%) died. Survival varied significantly by age group: mortality was highest among individuals aged ≥60 years (23.5%) and lowest among children under 12 (6.5%) (χ² = 61.7, *p* < 0.001). Gender did not show a statistically significant association with survival (*p* = 0.401). However, patients in disease-exposing occupations had lower mortality (9.5%) compared to those in non-disease-exposing roles (13.7%), with the difference reaching statistical significance (χ² = 8.15, *p* = 0.004). Rural patients had higher fatality rates (23.1%) than urban patients (12.7%) (χ² = 519, *p* < 0.001).

### 7.3. Estimated Comorbidities

Comorbidity data were not available in the primary dataset; however, estimates from the regional literature suggest that among EVD cases, the most common comorbidities were hypertension (25.4%), diabetes (14.0%), obesity (5.1%), and heart disease (3.9%). These estimates were used to approximate clinical risk within the sample. Table 2 provides the estimated comorbidities associated with infectivity related to EVD.

### 7.4. Occupational Exposure Among Infected Healthcare Workers

Among 438 healthcare workers with confirmed EVD infections, the most affected occupations included nurses (47.7%), hygienists (17.4%), and administrators (10.5%). Doctors accounted for 4.6% of infected HCWs, while laboratory technicians, students, and midwives also represented notable proportions. Table 3 provides the occupations most affected among healthcare workers during the Ebola outbreak.

### 7.5. Survival Analysis

Multivariate Cox regression revealed that **clinical status at admission** was the strongest predictor of mortality. Patients who were classified as “very sick” had an adjusted hazard ratio (HR) of **236.26** (95% CI: 33.18–1682.21; *p* < 0.001), compared to those with no symptoms. Patients in non-disease-exposing occupations had a significantly increased risk of death (HR: **1.75**, 95% CI: 1.33–2.31; *p* < 0.001), relative to those in disease-exposing occupations. Although comorbidity presence was associated with a higher unadjusted risk of death (HR: 3.05, *p* < 0.001), this association lost statistical significance after adjustment (HR: 1.17, *p* = 0.301). Age was a significant predictor in univariate analysis (HR per year increase: 1.02, *p* < 0.001) but not after multivariate adjustment (*p* = 0.062). Table 4 provides the overall survival (OS) analysis of treated Ebola cases.

## 8. Discussion

This commentary presents an analysis of 3477 confirmed Ebola virus disease (EVD) cases reported in the Democratic Republic of Congo (DRC) between 2014 and 2022. The data highlight significant progress in EVD management, with a notably lower case fatality rate (CFR) of 12.9%, a substantial reduction from the global historical range of 25% to 90% [1]. This improved outcome may reflect enhancements in the DRC’s outbreak response infrastructure, clinical management, and accumulated field experience across multiple epidemics.

Despite this encouraging trend, mortality remains strongly associated with specific clinical and contextual risk factors. The most powerful predictor of death was the patient’s condition at admission; those classified as “very sick” faced an exceptionally high hazard ratio (HR) of 236.26 (95% CI: 33.18–1682.21), reflecting both the severity of illness and likely delays in care-seeking. Patients in non-disease-exposing occupations also had significantly higher mortality, suggesting that occupational context—and, by extension, levels of risk awareness and access to early care—plays a critical role in outcomes.

### 8.1. Contextualizing the DRC’s Mortality Trends

Compared with previous outbreaks in West Africa, such as those in Guinea (66% CFR) and Sierra Leone (28%) during the 2014–2016 epidemic [2,3,4], the DRC’s lower CFR may indicate improved emergency preparedness and faster case detection [5]. However, these benefits appear unequally distributed. Over 91% of the cases in this cohort originated from Nord Kivu Province, underscoring regional disparities in both disease burden and access to healthcare services.

Occupational status served as a proxy for systemic exposure to EVD education, early warning systems, and health services. Individuals in non-clinical professions may face reduced awareness and fewer touchpoints with the health system, contributing to delayed care and higher risk of severe disease at presentation. Similar observations have been made in other settings where healthcare-focused risk communication failed to reach broader community groups [6].

### 8.2. Strengths of the Study

This analysis benefits from a large, real-world dataset spanning multiple outbreaks and regions, offering a rare longitudinal view of trends in EVD morbidity and mortality. The combination of descriptive, univariate, and multivariate survival analyses allowed for a nuanced understanding of both static (e.g., occupation) and dynamic (e.g., health status at admission) risk factors. The inclusion of literature-based estimates for comorbidities also provided a broader context, compensating for missing clinical details.

### 8.3. Limitations

Nonetheless, the study is subject to several limitations. The retrospective and observational nature introduces potential biases, particularly due to missing or inconsistent clinical records. Clinical severity classifications were likely based on subjective assessment, potentially varying across facilities. In addition, the lack of standardized virological, immunological, and treatment adherence data limits biological interpretation and reduces the capacity to assess intervention effectiveness.

Moreover, the occupational categories were broad and lacked granularity; further stratification (e.g., frontline clinical staff vs. support personnel) would offer more targeted insights. Regional differences in healthcare infrastructure were not directly analyzed but likely confound both presentation timing and outcomes. Finally, comorbidity data were inferred from secondary sources [7], which may not fully represent this cohort’s actual burden. Going forward, research should explore long-term outcomes among EVD survivors, including post-viral complications and socio-economic recovery. Prospective studies incorporating molecular diagnostics, treatment timelines, and behavioural data could yield more precise interventions. Moreover, qualitative research on stigma, trust, and healthcare utilization in outbreak-prone communities will be essential to ensure that response strategies are both evidence-based and locally grounded [8,9,10,11,12].

### 8.4. Public Health Implications

These findings underscore persistent vulnerabilities in the DRC’s outbreak response framework. While reduced mortality signals progress, the continued link between death and delayed presentation points to enduring structural barriers, ranging from geographic inaccessibility and weak referral systems to public mistrust. Comparisons with countries like Uganda, where decentralized care and rapid triage have improved outcomes, highlight the importance of system-wide resilience and preparedness [6].

To build a more robust response, especially in high-risk provinces like Nord Kivu, there is a pressing need for the following:

- Strengthened community-based surveillance to support early detection and intervention.
- Inclusive and culturally sensitive risk communication targeting populations beyond the healthcare sector.
- Sustained investment in decentralized diagnostic and referral infrastructure.
- Integration of epidemic preparedness into routine health system strengthening.
- It is recommended to prioritize targeted Ebola vaccination efforts in high-risk areas such as Nord Kivu, using the WHO-endorsed ring vaccination approach with the rVSV-ZEBOV-GP vaccine. This should include immunizing contacts of confirmed cases, their contacts, frontline health workers, and high-risk groups such as bushmeat hunters and EVD survivors, to prevent zoonotic transmission and resurgence. Ensuring rapid vaccine deployment, maintaining adequate stockpiles, and engaging communities through clear, culturally appropriate communication are essential to maximizing the impact of vaccination and preventing future outbreaks.

## 9. Conclusions

Recurrent EVD outbreaks in the DRC continue to exert a disproportionate toll on vulnerable populations, with mortality closely linked to clinical severity at presentation, occupational exposure, and systemic inequities. While the overall CFR has declined, preventable deaths persist, driven by delays in seeking care and unequal access to critical services. These trends signal the need for a paradigm shift: from reactive emergency response to proactive, community-anchored public health planning.

## Figures and Tables

**Table 1:** Socio-demographic and clinical characteristics of Ebola cases by outcome (N = 3477).

Variable	Category	Total N (%)	Survivors N (%)	Fatalities N (%)	χ²	*p*-Value
**Age (years)**	0–12	748 (21.5%)	699 (20.1%) (93.5%)	49 (1.4%) (6.5%)	61.7	<0.001
	13–19	500 (14.4%)	456 (13.1%) (91.2%)	44 (1.3%) (8.8%)		
	20–59	2076 (59.7%)	1755 (50.5%) (84.5%)	321 (9.2%) (15.5%)		
	≥60	153 (4.4%)	117 (3.4%) (76.5%)	36 (1.0%) (23.5%)		
**Gender**	Female	1693 (48.7%)	1461 (42.0%) (86.3%)	232 (6.7%) (13.7%)	1.83	0.401
	Male	1784 (51.3%)	1566 (45.0%) (87.7%)	218 (6.3%) (12.3%)		
**Profession**	Disease-exposing	632 (18.2%)	572 (16.5%) (90.5%)	60 (1.7%) (9.5%)	8.15	0.004
	Non-disease-exposing	2845 (81.8%)	2455 (70.6%) (86.3%)	390 (11.2%) (13.7%)		
**Location**	Rural	78 (2.2%)	60 (1.7%) (76.9%)	18 (0.5%) (23.1%)	519	<0.001
	Urban	3399 (97.8%)	2967 (85.3%) (87.3%)	432 (12.4%) (12.7%)		

**Table 2:** Estimated comorbidities among Ebola cases (Estimated N = 764). Based on literature-derived estimates for Ebola cases with known comorbidities.

Comorbidity	Estimated N	Prevalence (%)
Hypertension	194	25.4%
Diabetes	107	14.0%
Obesity	39	5.1%
Heart Disease	30	3.9%
Asthma/COPD	26	3.4%
Chronic Kidney Disease	7	0.9%
Active Tuberculosis	19	2.5%
HIV	12	1.6%

Estimates adapted from DRC and sub-Saharan African clinical literature (2020–2023).

**Table 3:** Occupations most affected among healthcare workers during the Ebola outbreak (N = 438). Healthcare workers with confirmed Ebola infections.

Occupation	N	Prevalence (%)
Nurse	209	47.7%
Hygienist	76	17.4%
Administrator	46	10.5%
Lab Technician	25	5.7%
Doctor	20	4.6%
Medical/Nursing Student	19	4.3%
Midwife	14	3.2%
Data Manager	10	2.3%
Pharmacist	4	0.9%
Physiotherapist	3	0.7%
Other	12	2.7%

Ebola vaccine uptake among HCWs in North Kivu, DRC (2021).

**Table 4:** Overall survival (OS) analysis of treated Ebola cases. HR = Hazard Ratio, 95% CI = Confidence Interval.

Characteristics	Category	n (%)	Univariate Analysis			Multivariate Analysis		
			HR	95% CI	*p*-Value	HR	95% CI	*p*-Value
**Comorbidity**	No	3210 (92.3)	Reference			Reference		
	Yes	267 (7.7)	3.05	2.41–3.87	<0.001	1.17	0.87–1.59	0.301
**Health Status**	No symptom (s)	222 (6.4)	Reference			Reference		
	Mildly sick	2676 (77.0)	3.46	0.48–25.09	0.219	3.46	0.48–25.11	0.219
	Very sick	579 (16.7)	244.63	34.37–1741.37	<0.001	236.26	33.18–1682.21	<0.001
**Exposed/non-exposed**	Disease-exposing activity	632 (18.2)	Reference			Reference		
	Non-disease-exposing activity	2845 (81.8)	1.46	1.11–1.91	0.006	1.75	1.33–2.31	<0.001
**Age**	Mean (SD) = 26.5 (16.1)	—	1.02	1.02–1.03	<0.001	1.01	1.00–1.01	0.062

## Data Availability

No new data were created or analyzed in this study. Data sharing is not applicable to this article.

## Abbreviations

**EVD**	Ebola Virus Disease
**HR**	Hazard Ratio
**CI**	Confidence Interval
**DRC**	Democratic Republic of Congo
**SD**	Standard Deviation

## References

[1] WHO Ebola Disease. https://www.who.int/news-room/fact-sheets/detail/ebola-virus-disease.

[2] Nyenswah T., Fahnbulleh M., Massaquoi M., Nagbe T., Bawo L., Falla J.D., Kohar H., Gasasira A., Nabeth P., Yett S. (2014). Ebola epidemic—Liberia, March–October 2014. MMWR Morb. Mortal. Wkly. Rep...

[3] WHO Ebola Response Team (2014). Ebola virus disease in West Africa—the first 9 months of the epidemic and forward projections. N. Engl. J. Med..

[4] Bah E.I., Lamah M.-C., Fletcher T., Jacob S.T., Brett-Major D.M., Sall A.A., Shindo N., Fischer W.A., Lamontagne F., Saliou S.M. (2015). Clinical presentation of patients with Ebola virus disease in Conakry, Guinea. N. Engl. J. Med..

[5] Ilunga Kalenga O., Moeti M., Sparrow A., Nguyen V.K., Lucey D., Ghebreyesus T.A. (2019). The ongoing Ebola epidemic in the Democratic Republic of Congo, 2018–2019. N. Engl. J. Med..

[6] Lamunu M., Olu O.O., Bangura J., Yoti Z., Samba T.T., Kargbo D.K., Dafae F.M., Raja M.A., Sempira N., Ivan M.L. (2017). Epidemiology of Ebola Virus Disease in the Western Area Region of Sierra Leone, 2014-2015. Front Public Health.

[7] Frieden T.R., Damon I., Bell B.P., Kenyon T., Nichol S. (2014). Ebola 2014—new challenges, new global response and responsibility. N. Engl. J. Med..

[8] Dhillon R.S., Kelly J.D. (2015). Community trust and the Ebola endgame. N. Engl. J. Med..

[9] Vinck P., Pham P.N., Bindu K.K., Bedford J., Nilles E.J. (2019). Institutional trust and misinformation in the response to the 2018–19 Ebola outbreak in North Kivu, DR Congo: a population-based survey. Lancet. Infect. Dis..

[10] Richardson E.T., Barrie M.B., Kelly J.D., Dibba Y., Koedoyoma S., Farmer P.E. (2016). Biosocial Approaches to the 2013-2016 Ebola Pandemic. Health Hum. Rights.

[11] Barry M., Traoré F., Sako F., Kpamy D., Bah E., Poncin M., Keita S., Cisse M., Touré A. (2014). Ebola outbreak in Conakry, Guinea: Epidemiological, clinical, and outcome features. Med. Mal. Infect..

[12] Fowler R.A., Fletcher T., Fischer W.A., Lamontagne F., Jacob S., Brett-Major D., Lawler J.V., Jacquerioz F.A., Houlihan C., O’dEmpsey T. (2014). Caring for critically ill patients with Ebola virus disease. Perspectives from West Africa. Am. J. Respir. Crit. Care Med..

